# A Case of Esmolol-Induced False-Positive Amphetamine Urine Drug Test

**DOI:** 10.7759/cureus.12429

**Published:** 2021-01-02

**Authors:** Jacob J Adashek, Arjun Khadilkar, Juan Enciso, Rishi Rane, Robby Wu

**Affiliations:** 1 Internal Medicine, University of South Florida (USF) Health, Tampa, USA; 2 Emergency Medicine, University of South Florida (USF) Health, Tampa, USA; 3 Cardiology, University of South Florida (USF) Health, Tampa, USA

**Keywords:** esmolol, false positive, atrial fibrillation, amphetamines, beta-blockers

## Abstract

False-positive urine drug screens can occur and lead to implicit bias. Confirmatory testing with gas chromatography/mass spectrometry can be performed. A morbidly obese patient with newly diagnosed atrial fibrillation spent multiple days in the cardiac intensive care unit (ICU) due to a false-positive test for methamphetamine. The patient was planned to undergo direct cardioversion with conscious-sedation anesthesia. His care was delayed because anesthesia was not comfortable administering sedatives in the setting of a positive urine drug screen for presumed methamphetamine use. Knowing that esmolol can cause a false positive on urine drug screen is imperative for delivering the best patient-centered care.

## Introduction

The urine drug screen (UDS) can be important in the management of patients who present to the emergency department (ED). Patients may often provide medical histories that are incongruent with their medication histories for a multitude of reasons: lack of medical literacy, guilt/shame, or malingering [1]. UDS testing allows for additional objective data to inform patient care but requires clinical reasoning and contextual interpretation. Although the UDS is still a controversial topic for overall quality and improvement in patient outcomes, this test is still one of the most utilized modalities for assessing potential complications of drug-drug interactions [2-3]. There are two common modalities for UDS: automated immunoassay testing and two-step confirmatory testing [4]. Generally, a drug or its metabolite is either detected or determined to be absent within a UDS without quantifying the concentration. An initial screening test can be confirmed with gas chromatography and mass spectrometry testing [5]. Immunoassay testing is more commonly used in point-of-care testing due to expedited results and cheaper costs compared to two-step confirmatory testing, which often requires additional send-out laboratory testing [5-6].

One major concern with UDS testing is false positives. In a retrospective study, roughly 11% (389 of 3,571) of samples tested positive for amphetamines or ecstasy on the initial immunoassay test but were confirmed negative by liquid chromatography-mass spectrometry, thus indicating a false-positive test [7]. This can occur through cross-reactivity of multiple commonly prescribed prescription medications, whose metabolites can lead to false-positive test results in conventional automated immunoassay UDS testing [6]. Medications such as labetalol, promethazine, methylphenidate, and trazodone have been implicated as causative agents of false-positive UDS results for amphetamines [6]. Additionally, the window of detection for amphetamines appears to be dose-dependent and related to factors including urine pH and the chronicity of use of the drug [8]. Unfortunately, false-positive testing can also lead to negative implicit bias of the patient and hinder trust and prejudice between physicians and nursing staff [9-10]. This case represents the first reported incident of a patient who tested positive on a UDS for amphetamines in the setting of an esmolol infusion.

## Case presentation

A 27-year-old male with class III obesity (body mass index (BMI): 69.81) presented with intermittent palpitations associated with lightheadedness, dizziness, and dyspnea for a month. He had no chest pain or any prior cardiac testing. The patient reportedly drank one to two alcoholic beverages on the weekends and adamantly denied any tobacco or recreational drug use. The patient had no prior hospitalizations, and his medical records were consistent with outpatient follow-up and only a previous prescription for methylprednisolone and azithromycin for the treatment of community-acquired pneumonia. When questioned repeatedly by several clinicians, the patient consistently stated he had not used any recreational drugs nor used cough suppressants, nasal decongestants, weight loss medications, attention-deficit/hyperactivity disorder medications, diabetes medications, or anti-depressant medications.

In the ED, an electrocardiogram (EKG) was performed and demonstrated atrial fibrillation with a rapid ventricular response with a heart rate of 175 beats per minute. Laboratory data were significant for an elevated brain natriuretic peptide of 316 pg/mL (normal < 100 pg/mL), and chest X-ray demonstrated an enlarged cardiac silhouette. Due to the presence of a mildly elevated D-dimer, a pulmonary angiogram was ordered and was found to be negative for an acute pulmonary embolus. The patient was placed on a continuous diltiazem infusion and heparin infusion for new-onset atrial fibrillation, and he continued to have sustained tachycardia with heart rates in the 150-160s. As part of the ED protocol for new-onset atrial fibrillation in patients under 60 years old, a UDS was ordered in the ED but was not performed, as the patient had been admitted to the cardiac intensive care unit and this order was lost in transit.

Later that evening, the patient was switched to continuous esmolol infusion and oral diltiazem. A transthoracic echocardiogram was performed and showed a severely reduced left ventricular ejection fraction of 30%-35%. The initial UDS was sent the following morning and resulted positive for amphetamines.

**Figure 1:**
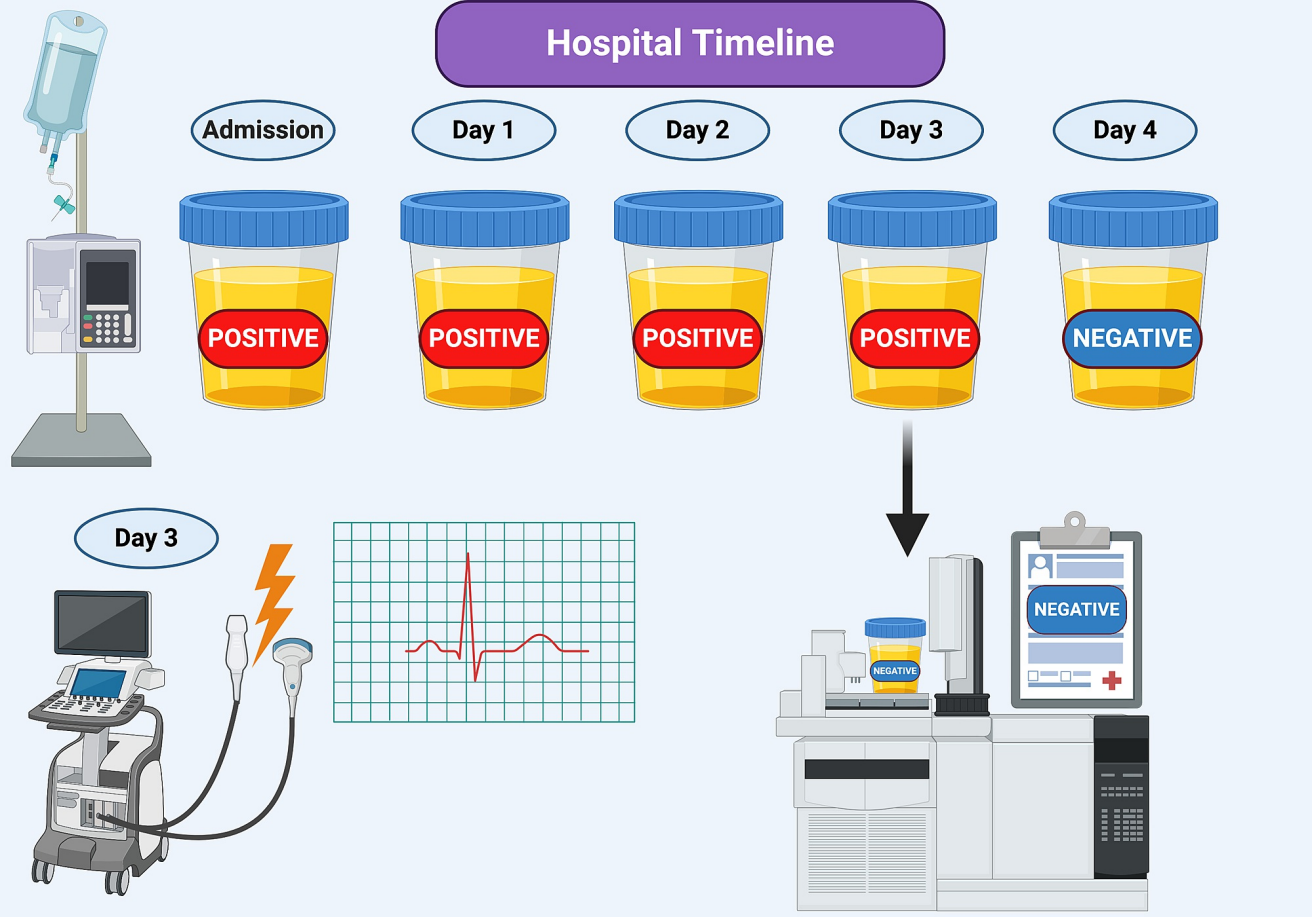
Hospital course of the patient with corresponding UDS UDS: urine drug screen

Due to the patient’s persistent arrhythmia, an electrophysiology consultation was placed. Given the patient’s newly identified cardiomyopathy, which was presumed to be secondary to uncontrolled atrial fibrillation, direct current cardioversion was recommended. Also, in light of the newly identified cardiomyopathy, medications were switched from esmolol infusion and oral diltiazem to amiodarone infusion and oral metoprolol succinate. In addition, anticoagulation therapy with warfarin was initiated for stroke prophylaxis. Prior to planned cardioversion, the anesthesiology service initially refused to consciously sedate the patient in the presence of positive amphetamines on his UDS. Subsequently, serial UDS were collected for two more days and continued to result positive for amphetamines. On hospital day three, despite a positive amphetamine result, the patient underwent a successful transesophageal echocardiogram and cardioversion with the restoration of normal sinus rhythm. A urine sample was sent out for gas chromatography/mass spectrometry confirmatory testing on day three, which resulted four days later as negative for amphetamine, methamphetamine, 3,4-methylenedioxyamphetamine, 3,4-methylenedioxymethamphetamine, and 3,4-methylenedioxymethamphetamine. He was continued on amiodarone for maintenance therapy. On hospital day four, UDS was ordered and resulted negative for amphetamines (Figure 1).

## Discussion

The UDS is an objective test that can either help negate or support a patient’s provided history, particularly on presentation to the ED. In a prospective cohort study between June 2012 and January 2013, 55 patients who presented with nausea or pain to an academic United States ED were interviewed after initial stabilization and all prescription medications, over-the-counter medications, and illicit drugs ingested for 48 hours prior to admission were documented [1]. The majority (69.1%) of self-reported patient histories were inconsistent with the UDS; only 17 of 55 patients had histories congruent with their UDS. Furthermore, 29.1% of patients (n = 16) had medications on the UDS that were not reported, including nine patients with unreported opioid medications [1]. This study represents a common problem faced by many emergency medicine physicians.

Inaccurate understanding of patients’ medication history, particularly illicit drug use, can have negative downstream effects including the inability for physicians to adequately educate patients about potential health risks, refer patients to appropriate detoxification programs, and identify life-threatening interactions such as serotonin syndrome [11]. Collectively, the miscommunication can lead to increased health care costs such as additional days in the hospital because of the inability to perform certain procedures due to the reluctance to administer timely anesthesia in the setting of a false positive amphetamine result [11-12]. Furthermore, false-positive testing can lead to negative patient bias and impact the patient relationship with the physician and nursing staff [9].

The phenomenon of false-positive UDS has been reviewed in the literature. Since 2000, there have been 62 published articles that include false-positive UDS testing for multiple drug classes [2,4]. A case series of three pregnant women demonstrated a false-positive UDS for amphetamine in the setting of labetalol for the management of hypertension [13]. This case described a specific labetalol metabolite, 3-amino-1-phenylbutane, which had a chemical composition that may potentially cross-react with a multitude of immunoassays and ultimately lead to false-positive UDS testing for amphetamines [13]. Although both labetalol and esmolol are beta-blockers, a similar cross-reactivity has not been described in the literature with esmolol.

**Figure 2:**
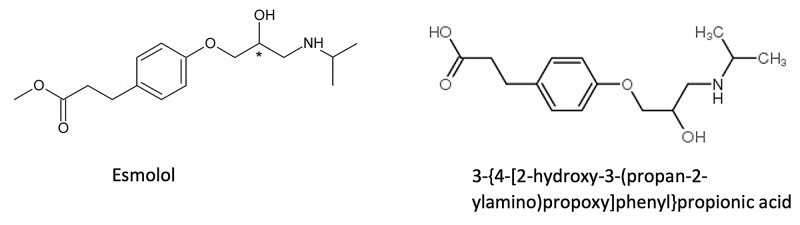
Chemical structure of esmolol and breakdown metabolite

Esmolol is a hydrolyzable ester moiety B1-selective antagonist, which undergoes rapid hemolysis in the blood, leading to fast-acting pharmacological effects. In vivo, esmolol can metabolize to the molecular component 3-{4-[2-hydroxy-3-(propan-2-ylamino) propoxy] phenyl}propionic acid, which has a 400 times lower activity to B1-receptors, as compared to its parent compound, esmolol (Figure 2). Theoretically, the positive amphetamine UDS during the patient’s hospitalization could have been as a result of the breakdown component or some unknown metabolite of similar composition. It is important to note the screening UDS remained positive for amphetamines while on esmolol infusion; ultimately, after the patient underwent cardioversion and was transitioned to amiodarone, the true UDS with subsequent confirmatory gas chromatography/mass spectrometry confirmatory resulted negative.

In this case, the patient received two different medications for rate control during new-onset atrial fibrillation: diltiazem and esmolol. Diltiazem, a non-dihydropyridine calcium-channel blocker, has been associated with false-positive UDS for lysergic acid diethylamide (LSD) but not with amphetamines [4]. The initial and subsequent UDS after the initiation of diltiazem infusion remained negative for phencyclidine, which is used to detect LSD. In addition, as seen in Figure 1, the same UDS, which tested positive for amphetamines on hospital day 3, was sent for confirmatory gas chromatography/mass spectrometry confirmation. Hypothetically, if the patient had surreptitiously consumed methamphetamines during hospitalization, both the urine drug screen and the confirmatory test would have resulted positive. This further supports a false-positive result from the UDS.

Another risk factor to consider in this case report is the patient’s morbid obesity (BMI: 69.81) with a relationship with new-onset atrial fibrillation. Although some case reports suggest a relationship between amphetamine use and new-onset atrial fibrillation, this patient had several other independent risk factors for new-onset atrial fibrillation [14-15]. Obese individuals have a 49% increased risk of developing atrial fibrillation as compared to non-obese individuals, with a positive correlation between increasing BMI and risk of developing atrial fibrillation [16]. Additionally, during this admission, the patient was diagnosed with obstructive sleep apnea, which is another independent risk factor for developing new-onset atrial fibrillation [17]. The patient’s new-onset arrhythmia, therefore, was thought to be more likely due to underlying comorbid conditions rather than amphetamine use.

## Conclusions

In this case study, positive amphetamine findings contributed to a delay in medical treatment and cardioversion, increased time in the cardiac intensive care unit, as well as overall lengthened hospital stay. Patients may suffer other negative consequences, including being subjected to implicit bias, mistrust, and prejudice by providers and staff. It is important to educate providers about the potential cross-reactivity of specific medications with UDS, particularly beta-blockers and amphetamines. Through this knowledge, clinicians may have a better clinical judgment in the interpretation of UDS leading to better and more efficient outcomes and becoming better practitioners.
